# MinePath: Mining for Phenotype Differential Sub-paths in Molecular Pathways

**DOI:** 10.1371/journal.pcbi.1005187

**Published:** 2016-11-10

**Authors:** Lefteris Koumakis, Alexandros Kanterakis, Evgenia Kartsaki, Maria Chatzimina, Michalis Zervakis, Manolis Tsiknakis, Despoina Vassou, Dimitris Kafetzopoulos, Kostas Marias, Vassilis Moustakis, George Potamias

**Affiliations:** 1 Computational BioMedicine Laboratory (CBML), Institute of Computers Science (ICS), Foundation for Research and Technology-Hellas (FORTH), Heraklion, Crete, Greece; 2 School of Electrical and Computer Engineering, Technical University of Crete, Greece; 3 Department of Informatics Engineering, Technological Educational Institute of Crete, Greece; 4 Institute of Molecular Biology & Biotechnology, FORTH, Heraklion, Crete, Greece; 5 School of Production Engineering & Management, Technical University of Crete, Greece; Johns Hopkins University, UNITED STATES

## Abstract

Pathway analysis methodologies couple traditional gene expression analysis with knowledge encoded in established molecular pathway networks, offering a promising approach towards the biological interpretation of phenotype differentiating genes. Early pathway analysis methodologies, named as gene set analysis (GSA), view pathways just as plain lists of genes without taking into account either the underlying pathway network topology or the involved gene regulatory relations. These approaches, even if they achieve computational efficiency and simplicity, consider pathways that involve the same genes as equivalent in terms of their gene enrichment characteristics. Most recent pathway analysis approaches take into account the underlying gene regulatory relations by examining their consistency with gene expression profiles and computing a score for each profile. Even with this approach, assessing and scoring single-relations limits the ability to reveal key gene regulation mechanisms hidden in longer pathway sub-paths. We introduce MinePath, a pathway analysis methodology that addresses and overcomes the aforementioned problems. MinePath facilitates the decomposition of pathways into their constituent sub-paths. Decomposition leads to the transformation of single-relations to complex regulation sub-paths. Regulation sub-paths are then matched with gene expression sample profiles in order to evaluate their functional status and to assess phenotype differential power. Assessment of differential power supports the identification of the most discriminant profiles. In addition, MinePath assess the significance of the pathways as a whole, ranking them by their p-values. Comparison results with state-of-the-art pathway analysis systems are indicative for the soundness and reliability of the MinePath approach. In contrast with many pathway analysis tools, MinePath is a web-based system (www.minepath.org) offering dynamic and rich pathway visualization functionality, with the unique characteristic to color regulatory relations between genes and reveal their phenotype inclination. This unique characteristic makes MinePath a valuable tool for in silico molecular biology experimentation as it serves the biomedical researchers’ exploratory needs to reveal and interpret the regulatory mechanisms that underlie and putatively govern the expression of target phenotypes.

## Author Summary

It is generally recognized that using different sources of information and knowledge is better than just using a single source. This is most profound in the post-genomics era. On one hand, the advent of genomic high-throughput technologies realized by DNA microarray and next generation RNAseq technologies enabled a ‘systems level analyses’ by offering the ability to measure the expression status of thousands of genes in parallel. On the other, molecular pathway networks depict the interaction of DNA segments during the transcription of genes into mRNA. The prominent and vital role of pathways in the study of various biology processes is a major sector in contemporary biology research. We introduce MinePath, a pathway analysis methodology that amalgamates information and knowledge from gene expression profiles and molecular pathways. The novelty of MinePath resides in its ability to target not just the genes involved in the pathways, as most of existing methodologies and tools do, but directly their interrelations and interactions. With this approach, the regulatory machinery that putatively governs and guides the expression of disease phenotypes can be explored and revealed.

## Introduction

Gene expression profiling via high throughput technology, either in the form of microarrays or in the form of next generation sequencing (NGS) and the subsequent quantitative measurement of RNA abundance in bio-samples, has generated (and continuous to generate) mass gene expression data streams for a number of targeted phenotypes and diseases. After the early years of microarray technology several research efforts were devoted towards the improvement of the quality of gene expression profiling protocols as well as for the standardization of the generated data, with the respective NGS based gene expression quantification to follow a similar progress [1]. On one hand, the selection of the most relevant features (gene transcripts), and the discovery of (diagnostic and prognostic) predictive biomarkers seems to have reached a mature state. On the other hand, the reliability and robustness of the induced biomarkers and predictive models as well as the translation of these models into clinical practice and the devise of respective trustworthy clinical decision-making scenarios remain open [2],[3]. The problem, already known and addressed by the machine learning and data mining research community, signifies domain dimensionality reduction denoted as feature selection [4],[5]. Initial expectation was that high throughput technology would reveal specific gene co-expression patterns for various phenotypes, but the utility of gene expression profiling seems to be bound to a number of limitations, mainly due to the complexity of the individual variations as well as the heterogeneity associated with the selected features and the induced gene signatures [6],[7],[8],[9].

In the last years, the bioinformatics research community has focused on more enhanced gene selection methods that amalgamate high-throughput gene expression measurements with knowledge from other bio-sources such as molecular pathways. As it is stated in [10], page 1: “*Given the functional interdependencies between the molecular components in a human cell*, *a disease is rarely a consequence of an abnormality in a single gene*, *but reflects the perturbations of the complex intracellular and intercellular network*”. In this sense, a network focus enables a more effective inference of key transcriptional changes, which are related to the specific phenotypes, by examining multiple downstream (or cross-talk) effectors of the target. The reached conclusion states that there is progress towards a reliable network-based approach to disease modelling, and at the same time it stresses the fact that progress is currently limited by the incompleteness of the available interactome map and the inadequacies in the existing pathway analysis methodologies and tools [10].

**Fig 1:**
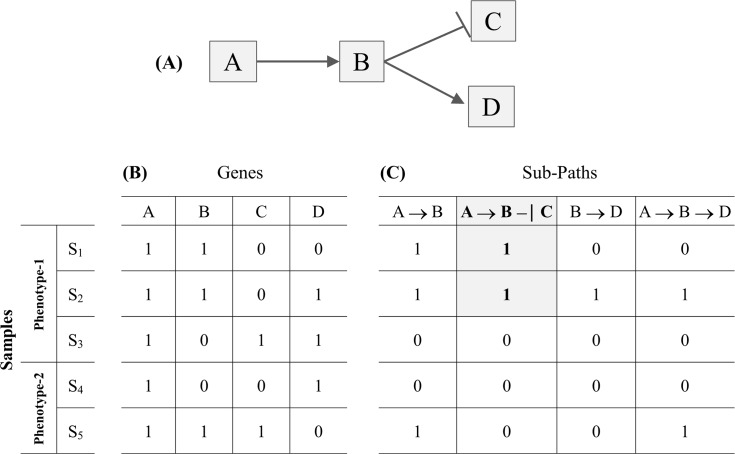
Limitations of analyzing solely gene expression profiles. (A) A dummy pathway. (B) The input (artificial) gene expression profile. (C) Functional status of sub-paths–the shaded cells indicate that sub-path A **→** B–| C is functional in the corresponding samples.

To reveal the problem and a way-out solution that also guided our research, Fig 1 provides an indicative example of the gene-selection limitations when analyzing solely gene expression data. Consider a dummy pathway that involves four artificial genes A, B, C and D (Fig 1A). Following a discretized setting, the gene expression levels are assigned to the binary values ‘1’ and ‘0’ meaning that the gene for the respective sample is up-regulated or down-regulated, respectively. The ‘→’ symbol represents and denotes an activation or expression relation that receives the following semantic interpretation: up-regulation of the source gene (i.e., taking the binary value ‘1’) makes its target gene also up regulated. The ‘–|’ symbol denotes an inhibition relation, i.e., up-regulation of the source gene makes its target gene down regulated (i.e., taking the binary value ‘0’). Note that the inhibition relation could be also considered as functional in the case where the inhibitor gene is down-regulated and the target gene is up-regulated (more about this ‘dual’ interpretation of the inhibition relation can be found in sections ‘From gene sets to topology and regulatory pathway machinery’). The input gene expression profile involves the four artificial genes (columns) and five samples (rows), with samples S_1_, S_2_, S_3_ assigned to Phenotype-1 and samples S_4_ and S_5_ to Phenotype-2 (Fig 1B). Looking at the gene expression profiles we may observe that no sole gene or group of genes can (perfectly) differentiate between the two phenotypes. Inducing a decision-tree with input this artificial binary gene expression dataset could prove it, i.e., all the branches of the induced tree conclude to conflicting phenotype assignments. In contrast, molecular pathways encompass additional biological features and knowledge–from the topology of the respective pathway network to the underlying gene regulatory relations (i.e., expression, activation, inhibition etc.), that may efficiently address the relevant gene selection barriers. In particular, gene interaction knowledge solves the major problem of conflicting constrains when two significantly up-regulated genes increase the enrichment of the gene-set in expression data, even if one of the genes acts as inhibitor of the other. Fig 1C shows the functional status of four sub-paths that may be formed after decomposing the dummy pathway into all of its component sub-paths (including the overlapping ones). It can be observed that the second sub-path, A → Β –| C, matches the gene expression profiles of the Phenotype-1 S_1_ and S_2_ samples (shaded cells) and none of the Phenotype-2 ones. So, the sub-path could be considered as functional for samples S_1_ and S_2_ and non-functional for samples S_3_, S_4_ and S_5_. The regulatory fingerprint reflected by this sub-path could be considered to ‘govern’ the expression status of the involved genes, and in a way it presents a putative cause for the expression of the specific phenotype in two, from the total of three, Phenotype-1 samples. Moreover, the pathway sub-paths could take the place of descriptors on the basis of which highly predictive phenotype differential models (decision trees or other) could be induced and formed.

### MinePath: Towards phenotype differentiating sub-paths

MinePath aims to address and cope with the aforementioned traditional pathway analysis problems and overcome the gene-set oriented visualization limitations, i.e., what color should be assigned to a target gene when, for one phenotype it is activated by an activator source gene, and for another phenotype it is inhibited by another source gene. MinePath fully exploits the topology as well as the underlying pathway gene regulatory relations, including the type and direction of these relations. Having on our disposal the sub-paths resulting from the functional decomposition of a pathway and the gene expression data, MinePath proceeds to the identification of the sub-paths that functionally differentiate between the targeted phenotype classes. The aim is the identification of those sub-paths that exhibit a high differential power to discriminate between the expression profiles of samples assigned to different phenotypes.

Existing and widely utilized pathway databases provide pathways of proved molecular value. Relevant on-line public repositories contain a variety of information that includes not only the pathway network per se but also incorporate links and rich annotations for the respective nodes (genes) and edges (regulatory relations). In its current implementation MinePath utilizes the KEGG pathways repository (www.genome.jp/kegg/pathway.html) [11]. KEGG pathways are widely utilized as a reference knowledge base for understanding biological pathways and the function of respective cellular processes. MinePath reads pathways directly from their original KGML representation format (KEGG markup language; www.genome.jp/kegg/xml). It is also able to cope with the richer XGMML format (a graph XML representation schema, also utilized by Cytoscape, wiki.cytoscape.org/XGMML) and so, it could be easily extended to manage other relevant pathway resources like, BioCarta [12], ReActome [13], and Pathway Commons [14].

Here we have to note that protein regulation may occur in both translational and post-translational levels, and KEGG encompasses and reports both protein and expression changes. Even though MinePath cannot directly detect post-translational modifications, the quantitative relations due to differences in gene expression are a strong indicator of protein regulation, and thus identification of related sub-paths remains a powerful tool for the identification of biologically significant relations. More details on this in section ‘Results/MinePath and mutation-based/driven or post translational modifications’.

### From gene sets to topology and regulatory pathway machinery

The main goal of this paper is to present MinePath–a pathway analysis approach that directly utilizes and exploits the underlying pathway topology and regulatory machinery, and contrast it with respective state-of-the-art approaches. As a systematic review of pathway analysis methodologies is out of the scope of this paper, we refer the reader to relevant extensive reviews [15], [16], [17] and [18].

A number of recent pathway analysis methodologies take advantage and exploit the topology and the gene regulatory relations of pathways. Furthermore, some of the relevant tools implement and offer network visualization functionality in order to map and display the underlying regulation machinery of pathways. Based on a literature search we identified relevant pathway analysis methodologies and tools that are presented in Table 1. The identified state-of-the-art methodologies and tools are presented in a unified and standardized notation, which expose their common characteristics in terms of the pathway features that they tend to utilize in their core processes.

**Table 1:** Scopes and main features of pathway analysis methodologies and tools. Each pathway analysis methodology is reported and referred by the first author of the original publication where the pathway analysis methodology and/or tool was presented, followed by the respective reference (column 1); the systems with names in bold (DAVID, SPIA, PATHOME, GGEA and GSEA) are the ones with which the MinePath is compared (section ‘Results’). The publication year is reported in column 2; the third column reports the respective ‘Pathway DBs/schemas’: pathway databases and schemas supported by the tool, where (1) represents KEGG, (2) ReActome, (3) BioCarta, (4) Pathway Commons, (5) WikiPathways, (6) Panther DB, (7) NDEx/PID, (8) MSigDB, (9) sbml, (10) xgmml, (11) BioPax, (12) GPML; the next columns refer to the main functionality offered and the features utilized by the respective method, i.e., ‘G’: Genes, ‘T’: Topology, ‘R’: Regulation, ‘GS’: Gene Selection, ‘PS’: Pathway Selection, ‘SPS’: Sub-Path Selection, ‘W’: Web-based, ‘Vg’: Visualization support using genes color coding, ‘Vi’: Interaction features of the visualization, ‘Vr’: Visualization support using relations color coding.

#	Publication [Ref]	Publ. Year	PathwayDBs / schemas	G	T	R	GS	PS	SPS	W	Vg	Vi	Vr
1	Siu [19]	2009	1,3	✓			✓						
2	Wang [20]	2008	1	✓			✓						
3	Braun [21]	2008	1	✓			✓	✓					
4	Tai [22]	2007	1	✓			✓						
5	Sfakianakis [23]	2010	1	✓			✓						
6	Beltrame [24]	2009	1,2,3	✓			✓						
7	Clément-Ziza [25]	2009	1,11	✓	✓		✓				✓		
8	Smoot [26]	2011	11	✓	✓		✓				✓		
9	Cline [27]	2007	10,11,12	✓	✓		✓	✓			✓		
10	Zhang [28]	2011	11	✓	✓			✓			✓		
11	Ibrahim [29]	2011	1	✓	✓		✓	✓					
12	Glaab [30] [31]	2010–12	1,3,5,6	✓	✓		✓	✓		✓	✓		
13	Subramanian[32]-**GSEA**	2005	8	✓			✓	✓					
14	Draghici [33]	2003	1	✓				✓					
15	Rhodes [34]	2007	1,3	✓				✓					
16	Cavalieri [35]	2007	1,2,3	✓				✓					
17	Adewale [36]	2008	8	✓				✓					
18	Ma [37]	2010	1	✓				✓					
19	Kelley [38]	2004	1	✓				✓			✓		
20	Warde-Farley [39]	2010	2,4,7	✓			✓	✓				✓	✓
21	Nacu [40]	2007	1	✓			✓	✓					
22	Chen [41]—**DAVID**	2011	1,3	✓	✓			✓		✓	✓		
23	Ulitsky [42]	2010	1,8	✓	✓			✓			✓		
24	Alcaraz [43,44]	2011–14	11	✓	✓			✓			✓	✓	
25	Ideker [45]	2002	11	✓	✓			✓					
26	Farfán [46]	2012	1,2,3,5,6	✓	✓			✓		✓			
27	Wu [47]	2012	1,2,6,7	✓	✓			✓					
28	Martini [48]	2013	1,2,3,7	✓	✓			✓			✓		
29	Kazmi [49,50]	2008–10	1.3	✓	✓			✓					✓
30	Li [51]	2009	1	✓	✓			✓		✓	✓		
31	Xia [52]	2010	1	✓	✓		✓	✓			✓		
32	Tarca [53]—**SPIA**	2009	1	✓	✓	✓		✓	✓	✓	✓		
33	Judeh [54]	2013	1	✓	✓	✓	✓	✓			✓		
34	Vandin [55]	2011	1	✓	✓	✓	✓	✓					
35	Vaske [56]	2010	7, 12	✓	✓	✓		✓		✓	✓		
36	Nam [57]-**PATHOME**	2014	1	✓	✓	✓	✓	✓					
37	Geistlinger[58]-**GGEA**	2011	1	✓	✓	✓	✓	✓		✓			
38	MinePath	----	1, 10	✓	✓	✓		✓	✓	✓		✓	✓

Observing Table 1 a general remark concerns the pathway knowledge that is utilized by each methodology. A bunch of pathway analysis approaches (#1–8 in Table 1) focus on the identification of differentially expressed genes. These approaches ignore, and do not employ in their methodology the topology and the underlying regulatory relations with an exception of the 7^th^ and 8^th^ method/tool in Table 1 that take into account only the pathway topology. Another group of pathway analysis methods (#9–31 in Table 1) move one-step further trying to identify discriminant pathways, even if they do not fully exploit the underlying pathway regulatory machinery. As a general remark we may state that: (a) most of the current pathway analysis tools focus mainly on the pathway enrichment characteristics of the target genes, and (b) they compromise the connectivity in favor of computational simplicity since the topology and the type of pathway relations are ignored or under-represented [59].

It is generally recognized that in order to efficiently address and to overcome the statistical barriers in traditional gene selection methodologies, the pathway topology and the underlying gene interactions should be taken into account [60]. Even in its infancy, this approach is followed by most of the recent pathway analysis methodologies (#32–38 in Table 1). They present a promising alternative towards the identification of the hidden underlying regulatory machinery that putatively governs and explains the expression of specific phenotypes. Representative systems include GGEA [58], SPIA [53], TEAK [54], HotNet [55], Paradigm [56], and PATHOME [57]. Moreover, even if these methodologies exploit the underlying pathway regulatory machinery, they reside on ‘summing’ over the functional status of the pathway regulatory gene relations without considering the exact functional status of each pathway relation or sub-path. Most of these approaches generate overall pathway ranks, with an exception of GGEA and Paradigm that provide respective sub-path views.

A key-component in order to indicate the predictive sub-paths and their power to differentiate between the target phenotypes is the efficient visualization of the pathway analysis results, which unfortunately most of these systems do not support. This obstructs the inspection of results and limits the user exploratory potential. Systems such as KEGG Atlas/Mapper [61], WebGestalt [62], NetworkTrial [63], Graphite Web [64], AltAnalyze [65], ReactomeFIViz [66] and EnrichNet [67] visualize just the pathway genes using a color-coding schema to indicate the strength of a pathway relation. The same holds for TEAK and GGEA that use a color-coding schema to visualize just the functional status of genes, not the functional status of sub-paths. In its extended version, Paradigm [68], visualizes the altered status of genes in the pathway while EnrichmentBrowser [69] R package enables the application of a range of set-based and network-based enrichment methods and provides visualization of results.

With the gene-set oriented visualization approach the problem is apparent even for small sub-paths like the single inhibition relation A—| B (A inhibits B; A, B represent genes). The inhibition relation exhibits a ‘dual’ character and could be considered as functional in a specific sample in two cases: when A is up-regulated and B is down-regulated or, when A is down-regulated and B up-regulated. In the first case, up-regulation of the A inhibitor causes the down-regulation of B. In the second case, the down-regulation of the inhibitor in a sense ‘allows’ B to be expressed and up-regulated. So, each of the genes should be visualized with a different color (just to indicate its expression status). The situation becomes even more complicated when one has to visualize the phenotype inclination of an interaction, for example when an inhibition relation is functional just for one phenotype and not for the other. Coloring and visualizing the functional status of pathway relations seems a promising alternative, and this is the approach that MinePath adopts and follows (see sections ‘Functionality and visualization capabilities of MinePath‘ for more details on the MinePath visualization conventions and functionality). The MinePath web-application may be accessed by www.minepath.org.

## Results

### Validation of MinePath

#### Self-assessment: Predictive performance

In order to assess the predictive performance of MinePath we used three publicly available breast cancer (BrCa) gene expression datasets from the Gene Expression Omnibus (GEO) repository (www.ncbi.nlm.nih.gov/geo)) namely, GSE3494 [70], GSE2034 [71], and GSE7390 [72] (all from the U133A Affymetrix platform, and available in the MinePath repository). We utilized the mygene information server (mygene.info) [73] to annotate and assign the respective microarray probe-sets to KEGG (Entrez) gene identifiers. From the initial U133A 22283 probe sets, 20902 share at least one Entrez gene identifier. As a gene can be mapped to more than one Entrez identifier, the expression profiles of these genes are replicated for all the Entrez identifiers, resulting into 22645 probe-sets—Entrez-id paired features for the input gene expression profiles. In all datasets, the estrogen-receptor status (ER+ and ER-) of the samples is reported. GSE3494 contains 213 ER+ and 34 ER- sample cases; GSE2034 209 ER+ and 77 ER-; and GSE7390 134 ER+ and 64 ER- (samples with missing ER status information were removed). ER represents a critical BrCa characteristic, with the activation and the underlying regulatory machinery of various growth-promoting pathways to be considered as most critical for the molecular characterization and prognosis of BrCa (e.g., the ErbB signaling pathway).

MinePath was applied on the three BrCa/ER datasets using all the human (hsa) KEGG pathways for respective train vs. test validation experiments (a total of 299 in the latest KEGG update; the nine global pathways maps were not retrieved). The decomposition of all input pathways resulted into a total of 34844 sub-paths, with an average of 137 sub-paths per pathway. The results, produced using the Weka/SMO (SMO is Weka’s support vector machine/SVM implementation) induction algorithm, are summarized in Table 2.

**Table 2:** Predictive performance of MinePath on three BrCa/ER datasets Rows and columns refer to training and test data, respectively. Bold figures for ‘Average ACC’ indicate superior performance; ‘SP’–sub-path, ‘ACC’–prediction (on unseen/test data) accuracy, ‘AUC’–Area Under the Curve, ‘Average ACC’–average accuracy, i.e., the average accuracy of GSE3494 over GSE2034 (89.5%) and GSE7390 (89.9%) is 89.7%.

Discriminant SPs	GEO dataset	GSE2034		GSE3494		GSE7390		AverageACC%
		ACC%	AUC	ACC%	AUC	ACC%	AUC	
888	GSE2034	---	---	89.1	0.652	83.3	0.767	86.2
1111	GSE3494	89.5	0.863	---	---	89.9	0.767	**89.7**
891	GSE7390	69.2	0.785	92.3	0.782	---	---	80.8
Average: 963.3								

The predictive performance of the induced models, in terms of ACC/AUC, ranges from 69.2% / 0.785 (for GSE7390 vs. GSE2034) to 92.3% / 0.782 (for GSE7390 vs. GSE3494). On average (‘Average ACC’), the best accuracy, 89.7%, is achieved by GSE3494. The figures signify that the selected most discriminant sub-paths (963.3 at an average across all datasets) achieve moderate to good predictive performance over independent datasets.

**Fig 2:**
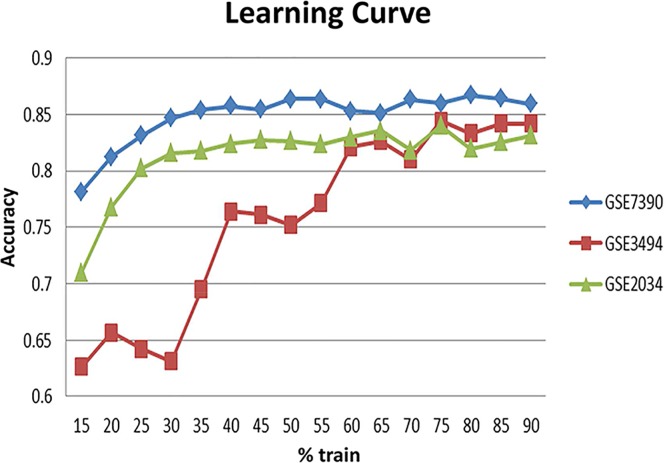
MinePath learning curve on three BrCa/ER datasets

In an attempt to assess the generalization power of the MinePath identified discriminant sub-paths we conducted the following experiment on each of the three BrCa datasets. Each dataset is split into training and test datasets starting from a 15% training split (the rest used for testing) and increasing it with 5% step. Note that the respective training and test data are discretized separately. Each step is iterated 100 times with respective random training-test splits, and the corresponding average accuracy figures are recorded. The result is the learning curve shown in Fig 2 (based on the average accuracy figures). The following observations could be made: (i) for the highly unbalanced GSE3494 dataset (213 ER+ vs. 34 ER- samples), about 75% of the data are needed in order to reach a stable accuracy performance (around 85%); (ii) for the more balanced datasets, GSE2034 (209 ER+ vs. 77 ER-) and GSE7390 (134 ER+ vs 64 ER-), stabilization of accuracy performance is achieved at about 35–40% of the data (around 83% and 87% accuracy for GSE2034 and GSE7390, respectively). The stabilized accuracy figures are comparative to the independent train-test experiment (reported in Table 2). The finding is indicative of MinePath’s ability to assess and select differential and discriminant sub-paths that does not suffer from overfitting, a situation that occurs in many traditional differential gene expression studies [74], [75].

**Fig 3:**
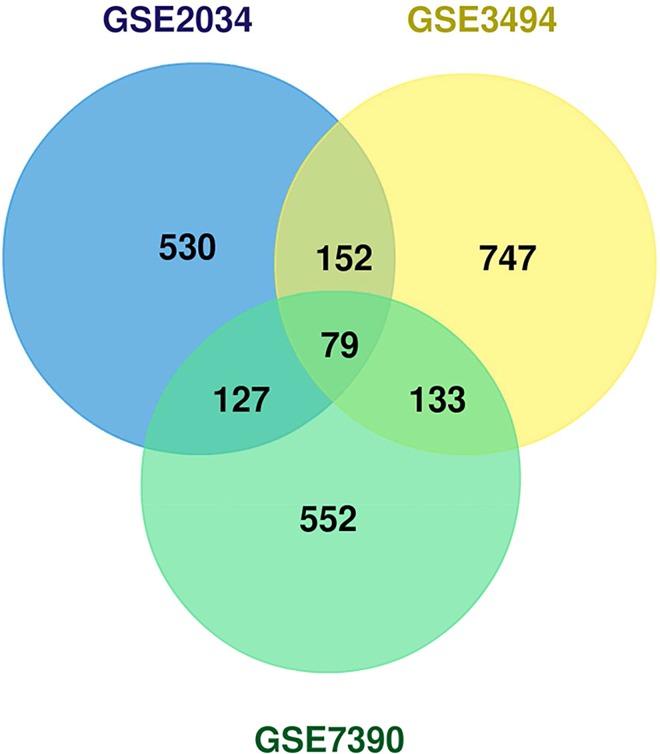
Venn diagram of the MinePath discriminant sub-paths that are shared among the three BrCa/ER datasets.

The number of discriminant sub-paths across datasets ranges from 888 (for GSE2034) to 1111 (for GSE3494); less than 3.5% of the total 34844 (decomposed) sub-paths. The Venn diagram in Fig 3 shows the overlap between the discriminant sub-paths across the three datasets. A total of 79 discriminant sub-paths are shared across the datasets, and these sub-paths involve 159 genes.

#### Self-assessment: Robustness analysis via permutation testing

It is important to verify that MinePath provides low false positive rates and delivers robust results, assessing if the discriminant sub-paths are not inferred just by chance. For this, we obtained permutation tests and computed respective false discovery rate (FDR) estimates. FDR assesses the expected proportion of statistically significant test results that are false positives, with FDR figures of less than 5% to indicate that the selected discriminant sub-paths are not selected by chance. The generation of respective permuted datasets achieved by shuffling the sample phenotype labels and preserving the balance of the phenotype assignments to the samples. For each of the three BrCa datasets (for the same ER+ vs. ER- differentiation task) and for each permutation we applied MinePath and identified the most discriminant sub-paths. The procedure was repeated 1000 times (resulting into an empirical null distribution of the selected sub-paths) and measured the FDR for the selected sub-paths on the original dataset. The GSE2034 selected sub-paths (888) exhibit FDR figures of less than 1%; the GSE3494 sub-paths (1111) exhibit FDR figures of less than 5% (with 125 out of them to exhibit FDR figures of less than 1%); and the GSE7390 891 sub-paths exhibit FDR figures of less than 5%, with 846 of them to exhibit FDR figures of less than 1%. The selected discriminant sub-paths for the GSE3494 dataset showed the worst performance, but still less than 5%. This could be attributed to the high imbalance of the GSE3494 dataset with respect to the targeted ER phenotypes, 213 ER+ vs. 34 ER-, with this unbalance to be reflected in the respective permutated datasets. In general, the permutation test results are indicative of the MinePath reliability in selecting robust phenotype differentiating sub-paths.

#### Self-assessment: MinePath for meta-analysis of gene expression studies

**Fig 4:**
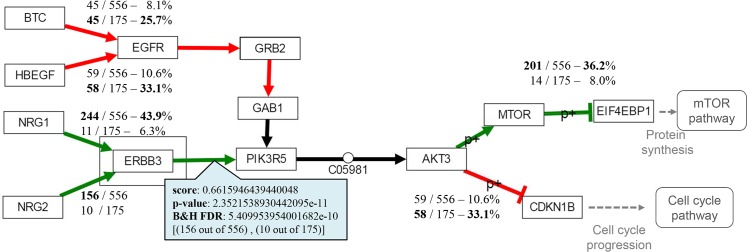
The MinePath identified sub-paths for the ‘3ER GSE2034-3494-7390’ merged dataset that discriminate between the ER+ and ER- phenotypes in the ErbB pathway. Edges colored in red indicate regulatory relations functional for the ER- phenotype, green for the ER+ phenotype, and black for relations that are functional for both phenotypes.

The ‘3ER GSE2034-3494-7390’ dataset (available in the MinePath repository) is a combination of the aforementioned three independent gene expression BrCa/ER studies (GSE2034, GSE3494 and GSE7390). Each dataset was discretized individually and the three discretized datasets were merged into the ‘3ER GSE2034-3494-7390’ dataset that contains 556 ER+ and 175 ER- sample cases. The merged dataset was analyzed in order to gain biological insights into the differentiation between the ER+ and ER- BrCa phenotypes. Application of MinePath on the merged dataset resulted into 26 pathways that exhibit p-values of less than 0.05 (see section ‘Identification of the phenotype differential sub-paths’ for the details on the MinePath process that assess the significant status of whole pathways). The ErbB pathway is in the top of the significant pathways (ranked fourth in the p-value ordered list of the total 299 targeted pathways, p-value ~ 0.000011). The ErbB pathway is known for its activation and over-expression in many cancers [76] and especially in BrCa [77]. The central role of ErbB pathway in the development of solid tumors, its availability to extracellular manipulation as well as its detailed understanding of the underlying biochemistry has made ErbB an attractive target for pharmacological intervention. Hence, ErbB signaling pathway is one of the most important pathways to explore. An edited and simplified (as resulted by various network rearrangements that are offered by respective MinePath visualization functionality) version of the ErbB pathway is shown in Fig 4.

Both ER phenotypes have extra-cellular origins (Fig 4). MinePath identified that the ER+ inclined path (in green) originates from the extra-cellular NRG1, NRG2 (neuregulin1, 2) growth factors that activate ERBB3 (erb-b2 receptor tyrosine kinase 3) viral oncogene, which in-turn activates PIK3R5 (phosphoinositide-3-kinase, regulatory subunit 5). The regulatory relation PIK3R5 → AKT3 (v-akt murine thymoma viral oncogene homolog 3) has no discriminant power since it is almost always functional for both phenotypes (functional in 726 out of the 731 input samples). MinePath visualizes relations and sub-path that are functional for both phenotypes (in black color) since such parts can link the gap between two discriminant sub-paths. Then, AKT3 activates MTOR (mechanistic target of rapamycin), which in-turn inhibits EIF4EBP1 (eukaryotic translation initiation factor 4E binding protein 1) that blocks ‘Protein synthesis’ events and initiates the mTOR pathway. Here we have to note that any other pathway analysis methodology would reject the PIK3R5 → AKT3 relation as being discriminant because it possesses no differential power and so, it would miss the extra-cellular triggering of the whole ER+ sub-path, NRG1,2 → ERBB3 → PIK3R5 → AKT3 → MTOR—| EIF4EBP1.

For the ER- phenotype, MinePath identified a discriminant sub-path that is also triggered from extra-cellular factors, HBEGF (heparin-binding EGF-like growth) or BTC (betacellulin), which in turn activate EGFR (epidermal growth factor receptor). Then, the receptor initiates the path GRB2 → GAB1 → PIK3R5 → AKT3 (note that PIK3R5 → AKT3 relation is shared with the aforementioned ER+ discriminant sub-path). At last, AKT3 inhibits CDKN1B (p27, Kip1, cyclin-dependent kinase inhibitor 1B) that blocks ‘Cell cycle progression’ events and initiation of the ‘Cell cycle’ pathway.

The aforementioned results are quite relevant to the BrCa/ER status. In [78] it is concluded that cell-cycle progression in ER- breast cancer cells can be regulated by nuclear factor Nf-kB with inhibitory activity being a potentially novel therapeutic agent for ER- breast cancer patients. Recent studies show the significant role of both ErbB3 and ErbB4 as alternative targets for the treatment of breast cancer patients suggesting a pan-ErbB inhibitor strategy that is able to interfere the cross-talk between the various ErbB receptors [79]. As noted in [80] “*the initial growth inhibitory effects of fulvestrant appear compromised by cellular plasticity that allows rapid compensatory growth stimulation via ErbB-3/4*. *Further evaluation of pan-ErbB receptor inhibitors in endocrine-resistant disease appears warranted*”. In addition, in [81] it is investigated whether induction of ErbB3 and/or ErbB4 may provide an alternative resistance mechanism to anti-hormonal action. The conclusion is that fulvestrant treatment is sensitive to the actions of the ErbB3/4 ligand HRGb1 (NRG1) with enhanced ErbB3/4-driven signaling activity and significant increases in cell proliferation.

#### MinePath and individualized medicine

One of MinePath’s visualization capabilities concerns the information of the single regulatory relations. By clicking an edge the user may get relevant information for the respective relation: polarity score, p-value, fdr, coverage (white rectangle in Fig 4). From the information we may observe that the extra-cellular pathway triggering relation NRG1 → ERBB3 covers 43.9% of the ER+ cases (244 out of 556), with the final sub-path inhibition relation MTOR—| EIF4EBP1 to cover 36.2% of them (210 out of 556). In other words, the whole ER+ differentiating sub-path from NRG1 to EIF4EBP1 is functional for a subset of ER+ cases, with the rest of cases to be covered by other sub-paths. This is a unique feature of MinePath that could be directly linked to the concept of individualized (or personalized) medicine, i.e., each individual (or groups of them) exhibit different molecular regulation characteristics that may influence and guide respective diagnostic, prognostic and therapeutic decisions. Towards this line of research MinePath has already been applied on the pediatric nephroblastoma cancer domain in an effort to reveal the role of targeted miRNAs in the disruption of engaged pathways [82].

### Comparing MinePath with state-of-the-art pathway analysis methodologies

The main goal of the comparison is to contrast MinePath with those state-of-the-art pathway analysis tools that utilize and exploit in their methodology the topology and/or the regulatory machinery of the pathways. Note that for most of these state-of-the-art tools, the comparison could not be performed directly on the level of the identified discriminant sub-paths since most of the methodologies do not report information per sub-path. However, we attempt such a comparison in order to assess to which extent the different approaches are able to identify and reveal biologically important regulatory pathway relations and sub-paths (see below the sub-section ‘Identification of discriminant cancer-related regulatory relation and sub-paths‘).

The heterogeneity in the pathway representation formats utilized by the various pathway analysis tools constraints their thorough comparison. This is also apparent for those tools that exploit in their methodology the topology and/or the regulatory relations of pathways. Either an alternation of pathway formats or an alteration of the underlying tools’ algorithmic process is required in order to accommodate the differences [17]. Various pathway analysis methodologies (e.g., Paradigm) support the BioPAX (level 2) standard (www.biopax.org) to represent pathways while MinePath and other (e.g., GGEA and SPIA) support the KGML KEGG standard (www.kegg.jp/kegg/xml). Therefore, we decided to conduct the comparison either with state-of-the-art pathway analysis systems that offer free implementations (e.g., GGEA and SPIA) or with systems for which the original publications report results on experiments that could be also conducted with MinePath (e.g., PATHOME, DAVID and SPIA).

### Comparing MinePath with SPIA and GGEA

#### Predictive performance

We compare MinePath with SPIA and GGEA utilizing again the aforementioned three BrCa/ER gene expression datasets (GSE3494, GSE2034 and GSE7390). The comparison experiments were conducted using the Enrichment Browser R/Bioconductor package (www.bioconductor.org/packages/release/bioc/html/EnrichmentBrowser.html)) that supports the GGEA and SPIA algorithms. Special functions of EnrichmentBrowser were utilized in order to download all human KEGG pathways. First we have to mention that the average execution time for the three datasets, taking into account all the (locally downloaded and retrieved) human KEGG pathways, was 330 seconds for SPIA, 353 for GGEA and 41 for MinePath, highlighting MinePath’s real-time operational efficiency.

SPIA and GGEA assess the consistency and compute the phenotype differential power of a sub-path based on the differential power of its constituent single regulatory gene relations. In contrast, MinePath computes directly the differential power of sub-paths, single or more complex ones. Due to this limitation, we had to conduct the comparison on the level of single-relations. So, we extracted all single-relations involved in the sub-paths that MinePath assessed as discriminant. In addition, since each gene is mapped to one or more transcripts, we created all the combinations of transcript relations for the respective gene relations. Furthermore, as SPIA and GGEA ignore the indirect KEGG relation, the respective transcript relations were ignored. Under the above assumptions a training single-relations binary matrix was formed for each method and for each of the three BrCa/ER dataset. The rows represented the single-relations selected as discriminant by each tool, the columns hold the samples, and each cell take the value ‘1’ when the relation is considered as functional for the respective sample, and ‘0’ otherwise. The Weka SMO/SVM induction algorithm was used in order to assess and compare the respective predictive performances. The results are presented in Table 3.

**Table 3:** Predictive performance of SPIA, GGEA and MinePath on three BrCa/ER datasets utilizing single-relations Rows and columns refer to training and test data, respectively. Abbreviations are the same with those used in Table 2; bold figures indicate superior performance.

**SPIA**								
**Discriminant SPs**	**GEO dataset**	**GSE2034**		**GSE3494**		**GSE7390**		**Average ACC%**
		ACC%	AUC	ACC%	AUC	ACC%	AUC	
569	GSE2034	—	—	86.2	0.549	79.3	0.708	82.8
414	GSE3494	79.4	0.637	—	—	71.7	0.571	75.6
469	GSE7390	80.1	0.691	**91.5**	0.815	—	—	**85.8**
Average:	484.0							
**GGEA**								
**Discriminant SPs**	**GEO dataset**	**GSE2034**		**GSE3494**		**GSE7390**		**Average ACC%**
		ACC%	AUC	ACC%	AUC	ACC%	AUC	
1006	GSE2034	—	—	87.9	0.559	**83.8**	0.770	**85.9**
693	GSE3494	**79.7**	0.722	—	—	**84.8**	0.881	**82.3**
525	GSE7390	77.6	0.630	89.9	0.632	—	—	83.8
Average:	741.3							
**MinePath**								
**Discriminant SPs**	**GEO dataset**	**GSE2034**		**GSE3494**		**GSE7390**		**Average ACC%**
		ACC%	AUC	ACC%	AUC	ACC%	AUC	
1271	GSE2034	—	—	**89.9**	0.657	68.7	0.516	79.3
2375	GSE3494	75.5	0.562	—	—	71.2	0.555	73.4
2917	GSE7390	**83.9**	0.722	87.0	0.740	—	—	**85.5**
Average:	2187.7							

SPIA exhibits the worst performance except for the GSE7390 vs. GSE3494 experiment (91.5%). GGEA performs better than MinePath in three of the six experiments: 83.8% (GSE2034 vs. GSE7390), 79.7% (GSE3494 vs. GSE2034), and 84.8% (GSE3494 vs. GSE7390). MinePath performs better than GGEA in two experiments: 89.9% (GSE2034 vs. GSE3494), and 83.9% (GSE7390 vs. GSE2034). The above performances achieved with a different number of selected relations for each tool, at an average 484, 741.3 and 2187.7 for SPIA, GGEA and MinePath, respectively. It is notable that MinePath includes a big number of single-relations compared to SPIA and GGEA. This is due to the fact that MinePath works and assesses the differential power of complex sub-paths instead of single-relations. As a consequent, the decomposition of each sub-path into its constituent relations results into a big number of single-relations. Table 4 shows the average predictive performance figures achieved by each method on each dataset. Rows ‘MinePath_sp’ and ‘MinePath_sr’ shows the performance figures of MinePath when the discriminant sub-paths and when the single-relations (involved in sub-paths) are used, respectively. MinePath provides comparative, and on average superior accuracy figures with the respective figures achieved by SPIA and GGEA, even though no statistical significant difference could be observed (using the Friedman's statistical test). The MinePath sub-paths (‘MinePath_sp’) approach exhibits the best performance for two datasets, 86.2% (GSE2034) and 89.7% (GSE3494), with SPIA to exhibit the best performance for just one dataset, 85.8% for GSE7390. At an average, the original ‘MinePath_sp’ approach achieves the best performance (85.6%) compared with ‘MinePath_sr’ (79.4%), SPIA (79.9%) and GGEA (84.1%).

**Table 4:** Average predictive performance of ‘MinePath_sp’, ‘MinePath_sr’, SPIA and GGEA. The best accuracies from Tables 2 and 3 are shown; ‘MinePatgh_sp’–MinePath using discriminant sub-paths; ‘MinePath_sr’–MinePath using the single-relation involved in the discriminant sub-paths (bold figures indicate superior performance).

Methodology	GSE2034	GSE3494	GSE7390	Average ACC%
	ACC%	ACC%	ACC%	
MinePath_sp	**86.2**	**89.7**	80.8	**85.6**
MinePath_sr	79.3	73.4	85.5	79.4
SPIA	82.8	75.6	**85.8**	79.9
GGEA	85.9	82.3	83.8	84.1

#### Identification of discriminant cancer-related regulatory relation and sub-paths

The three pathway analysis methodologies are based on the assessment of the differential power of pathway gene single-relations. MinePath computes a rank for the whole sub-path in order to select the most discriminant sub-paths, while SPIA and GGEA provide (binary) consistency scores for single-relations, with ‘1’ to be assigned to a single-relation found to be consistent with the expression status of the genes involved in the specific relations, and ‘-1’ otherwise. To make the comparison feasible we consider and interpret the consistency of single-relations as equivalent to the single-relations involved in the sub-paths found as discriminant by MinePath.

For the comparison we target the p53 pathway using the BrCa/ER GSE3494 dataset, as this is the only pathway shared as significant between the three methodologies. The differential power (with respect to ER+ vs. ER- phenotypes) of the p53 pathway is excepted to be high since 91% (31 out of the 34) of the ER- samples are characterized as p53-mutated, in contrast to the 19% (41 out of the 213) for the ER+ samples [71] (the mutation information is reported in the respective GSE3494 clinical data). The p53 signaling pathway is widely recognized as a key cancer related pathway [83], with ERbeta to present a therapeutically critical target for the decrease of the survival of p53-defective cancer cells after DNA damage [84]. It is also known that p53 is a major mediator for chemotherapy therefore, understanding the crosstalk between p53 and ER signaling may provide important clues to improve current BrCa treatment strategies. Even if various BrCa studies have given conflicting results, the strong correlation between ER factors and p53 is generally acceptable, with p53 to be considered as primarily wild-type in ER+ and mutated in ER- BrCa phenotypes [85].

**Fig 5:**
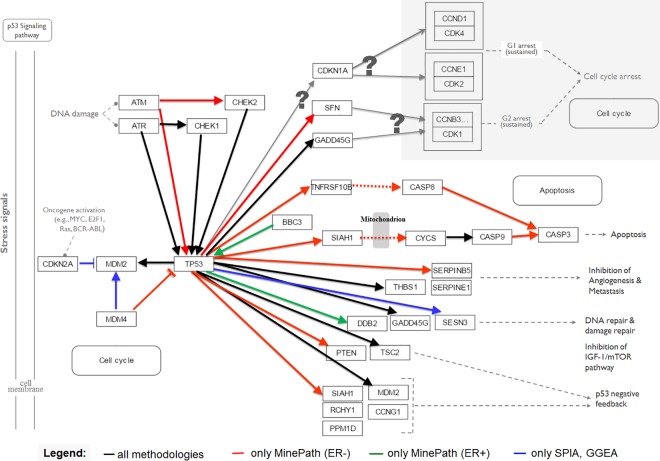
Contrasted SPIA, GGEA and MinePath results for the p53 pathway using the GSE3494 ER+ vs. ER- dataset. The legend shows the meaning of the edge colors used to contrast between the results produced by the three pathway analysis methodologies.

MinePath identified 37 discriminant sub-paths, 5 for ER+ and 32 for ER-; SPIA and GGEA identified 49 discriminant single-relations, 30 consistent and 19 inconsistent. For the specific dataset and the p53 pathway, the results of SPIA and GGEA are completely in agreement. Fig 5 presents and contrasts the results produced by SPIA, GGEA and MinePath. Different direct edge (activation/expression or inhibition) colors are used to contrast between the different methodologies. Black edges represent relations identified as discriminant by all methodologies (i.e., found as consistent by SPIA and KEGG, and discriminant for MinePath; note that SPIA and GGEA induce the same relations as consistent). Red and green represent relations identified as discriminant only by MinePath for the ER- and ER+ phenotypes, respectively, while blue represents relations identified as consistent only by SPIA and GGEA.

Examining Fig 5 the following observations could be made:

1. Upstream of tp53: First, DNA damage response (DDR) patterns are mainly controlled by two signaling relations, ATM → CHEK2 and ATR → CHEK1, each with its distinct role in cancer evolution and therapy [86]. MinePath is the only among the three methodologies that identifies as discriminant the ATM → CHEK2 relation, with CHEK2 to be considered as a susceptibility gene for inherited BrCa, also related with the mutation status of the well-known BrCa susceptibility genes BRCA1 and BRCA2 [87]. We also note that the ATM → CHEK2 relation is considered as discriminant for the ER- phenotype, a finding that is in agreement with the fact that many ER- patients show less sensitivity to DDR agents [88] exhibiting also much higher expression levels of ATM [89]. Second, MinePath is also the only methodology that reveals the role of MDM4 as an important inhibitor of p53 (the colored red MDM4—| TP53 inhibition relation in Fig 5), with SPIA and GGEA being unable to establish this relation as discriminant. It is demonstrated that MDM4 (or MDMX) is highly expressed in ductal epithelial breast cells and its down-regulation is sufficient to significantly mitigate tumor growth and progression, a fact that suggest the targeting of MDMX as an attractive strategy for the treatment of BrCas that express wt p53 (~70% of all cases) [90].
2. Downstream of p53: First note that none of the three pathway analysis methodologies were able to identify sub-paths or single-relations that guide to cell cycle arrest events (top-right shaded area, with the ‘?’ sign to indicate that the involved single-relations found as non-discriminant). According to literature, clinical-trials highlight the role of the involved cyclin-dependent kinases CDK4/6 complexes in BrCa, with their inhibition to present promising targets for BrCa progression-free survival, especially for the BrCa ER+ phenotype [91]. Second, MinePath is the only methodology that indicates BBC3 or PUMA as up-regulator of p53 (BBC3 → TP53 activation relation in green). PUMA is an estrogen target gene that is vastly down-regulated in response to estrogen in BrCa cell lines, and it is up-regulated by tamoxifen treatment. In addition, low expression of PUMA is significantly associated with BrCa-specific death and worse tamoxifen treatment outcome [92]. Third, MinePath is also the only methodology that induces as discriminant the sub-paths that guide to Apoptosis. Two such paths are identified, the extrinsic path TP53 → TNFRSF10B/TRAIL/DR5 → CASP8/FLICE → CASP3 ––> Apoptosis. TNFRSF10B (or TRAIL, DR5) is a TNF-related apoptosis-inducing ligand that possesses the unique capacity to induce apoptosis selectively in many cancer cells *in vitro* and *in vivo*, with CASP8 (or FLICE) to be recruited for downstream death signaling induction which is, in turn, sufficient to directly and fully activate the effector CASP3, resulting in apoptosis. A number of preclinical studies demonstrated the robust anticancer activity of TRAIL-receptor agonists and the stimulation of the extrinsic apoptosis pathway is bound to be more effective than chemotherapy for treating cancers with TP53 mutations [93]. Furthermore, with respect to the intrinsic sub-path TP53 → SIAH1 → CYCS → CASP9 → CASP3 ––> Apoptosis, a path that passes through SIAH1, it is known that up-regulated SIAH1 acts as a tumor suppressor [94], [95].

#### Identification of biology important pathways

**Fig 6:**
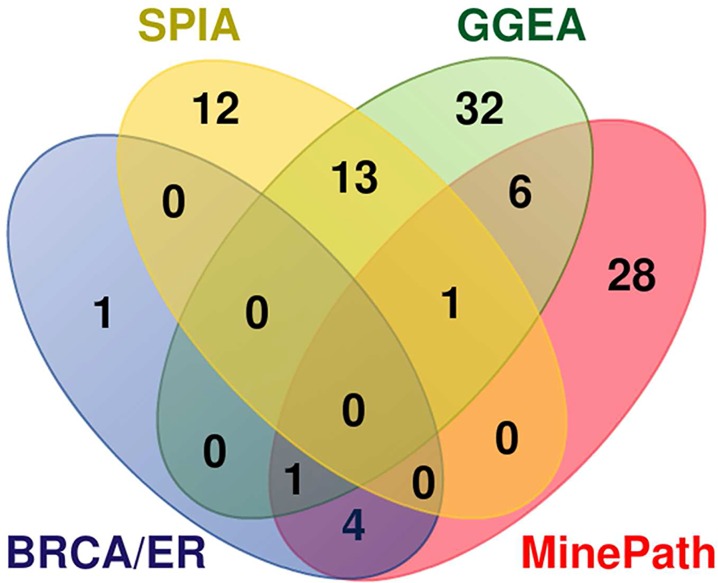
Venn diagram of the selected (significant) pathways shared among SPIA, GGEA, MinePath and the six significant BrCa/ER pathways

SPIA and GGEA compute the significance of a whole pathway based on the differential power of the involved genes. MinePath utilizes the pathway sub-paths (functional or not) in order to assess the differential power and significance (p-value) of the whole pathway. In this mode MinePath may be directly compared with SPIA and GGEA on the pathway level. Here we should notice that MinePath assesses the significance of pathways not on the basis of the involved genes but on the functional status of sub-paths in the pathways. In this respect we could say that MinePath comprises a sub-path set enrichment analysis methodology, just in analogy with gene set enrichment analysis methodologies (see section ‘ Identification of the phenotype differential power of sub-paths‘ / ‘Assessment of pathway significance’ for more details). The comparison is performed by targeting a set of known ER-related pathways. Various molecular pathways mediate endocrine resistance and evaluated for BrCa/ER therapeutic targets. The review in [96] reports the growth factor receptor signaling pathways as basic targets, as these factors are implicated in the development of endocrine resistance in the management of ER status. A total of six pathways are reported as significant: HER2 (ErbB) that causes endocrine resistance through crosstalk with ER; PI3K–AKT which is augmented in long-term estradiol-deprived cell-lines that have developed endocrine resistance; JAK–STAT which is activated by HER family receptors; Ras–Raf/MEK–ERK (engaging the MAPK and RAS KEGG pathways) that plays a role in HER receptor family-mediated endocrine resistance; and mTOR which is inhibited. For GSE2034, SPIA identified 17 significant pathways, GGEA 44 and MinePath 26, without a common pathway shared among the three methods. For GSE3494, SPIA identified 12 significant pathways, GGEA 25 and MinePath 17, with just the p53 pathway shared among the three methodologies. For GSE7390, SPIA identified 12 significant pathways, GGEA 21 and MinePath 19, with no common pathway among the three methodologies. The significant pathways shared among the three methodologies, and the list of the six significant BrCa/ER pathways are shown in the Venn diagram of Fig 6. SPIA shares no significant BrCa/ER pathways, GGEA share just one (JAK-STAT), and MinePath shares five of them: JAK-STAT (also shared with GGEA), ErbB, MAPK, RAS and mTOR. MinePath selected ErbB and MAPK pathways as significant in all three BrCa/ER datasets. The findings are suggestive for the ability of MinePath to identify important and biology relevant pathways.

### Comparing MinePath with PATHOME, DAVID and GSEA

PATHOME is one of the most recent pathway analysis tools that compute the differential power of regulatory relations in order to assess the phenotype differential significance of the whole pathway. In the system’s original publication [57], PATHOME was compared with two well-known pathway analysis tools, namely GSEA and DAVID. GSEA (Gene Set Enrichment Analysis, www.broadinstitute.org/gsea) follows a gene-set enrichment methodology in order to identify statistically significant phenotype differentiating gene-lists by assessing their functional enrichment in targeted pathways [32]. DAVID (Database for Annotation, Visualization and Integrated Discovery, david.ncifcrf.gov) is a widely utilized web-based environment that offers a set of functional annotation tools to reveal, assess and comprehend the underlying biological meaning of genes [59]. The comparison was made on the basis of a public Gastric Cancer (GC) gene expression dataset (GSE13861, with the Illumina HumanWG-6 v3.0 expression beadchip; it includes 65 primary GC frozen tissue samples and 19 normal appearing gastric tissue samples). Gastric cancer is the second leading cause of cancer-related death worldwide with most of the patients to receive similar treatment, typically surgery followed by chemotherapy, as there are no reliable biomarkers to optimize therapy [97]. For the comparison we used a reference standard of nine cancer-related pathway categories as reviewed by Vogelstein and Kinzler in [83]. Each pathway category refers to various single KEGG pathways (from a total of 15): HIF1 (mTOR/hsa04150, Pathways in cancer/hsa05200, Renal cell carcinoma/hsa05211), p53 (hsa04115), RB (Cell cycle/hsa04110), Apoptosis (Apoptosis/hsa04210), GLI (Hedgehog/hsa04340), APC (Wnt/hsa04310), RTK (ErbB/hsa04012, Pathways in cancer/hsa05200), SMAD (TGF-β/hsa04350) and PI3K (ErbB/hsa04012, Pathways in cancer/hsa05200, mTOR/hsa04150, MAPK/hsa04010, Insulin/hsa04910, Focal adhesion/hsa04510, Chenokine/hsa04062, VEGF/hsa04370).

In the original publication of PATHOME the authors do not provide access to the tool or the source code. We conducted the same experiment with MinePath (i.e., using the same dataset and all the KEGG human pathways) in order to compare with the results reported in [57]. Furthermore, even though PATHOME computes the differential power of sub-paths in order to assess the significance of the whole pathway, in the original publication only the significant pathways are reported. Under the aforementioned restrictions, the comparison is limited on the identified significant pathways. The significant pathways were identified on the basis of FDR (as reported in the original publication)–FDR < 0.05 for PATHOME and MinePath, (Benjamini) FDR < 0.3 for DAVID, and FDR (q-value) < 0.3 for GSEA that results into 27 selected significant pathways for PATHOME, 17 for GSEA, 15 for DAVID and 28 for MinePath. The comparison results are summarized in Table 5. MinePath identified as significant 5 out of the 15 reference cancer-related single KEGG pathways (MAPK, P53, mTOR, Wnt, Focal adhesion) that cover 4 out of the 9 reference standard cancer-related pathway categories (PI3K, P53, HIF1, APC); PATHOME identified as significant 6 out of the 15 reference cancer-related KEGG pathways (MAPK, Chenokine, Wnt, Focal adhesion, Insulin, Pathways in cancer) that also cover 4 out of the 9 reference standard cancer-related pathway categories (PI3K, APC, HIF1, RTK); DAVID identified as significant just one KEGG pathway (Focal adhesion) that covers just one cancer-related pathway category (PI3K); and GSEA just one KEGG pathway (Cell cycle) that covers just the RB/Cell cycle pathway category. Furthermore, the authors reported that five genes, WNT5A, VANGL1, SFRP2, FZD1 and PLCB1 are up-regulated in GC cases. Note that the APC/Wnt pathway is already validated as a pathway associated to gastric cancer [98].

**Table 5:** Comparative results for PATHOME (P), DAVID (D), GSEA (G) and MinePath (MP) in terms of selected pathways with reference to nine gold standard cancer-related pathway categories (see text for more details); PI3K –phosphoinositide-3-kinase; RTK–protein-tyrosine kinase; RB–retinoblastoma; HIF1 –hypoxia inducible factor; APC–antigen-presenting cell; GLI–glioma-associated oncogene; ‘✔’ identified as significant.

Cancer PW categories	KEGG PW code	Pathway name	PATHOME	DAVID	GSEA	MinePath
PI3K	hsa04010	MAPK signaling	✔			✔
PI3K, RTK	hsa04012	ErbB signaling				
PI3K	hsa04062	Chemokine signaling	✔			
RB (Cell cycle)	hsa04110	Cell cycle			✔	
P53	hsa04115	P53 signaling				✔
HIF1, PI3K	hsa04150	mTOR signaling				✔
Apoptosis	hsa04210	Apoptosis				
APC	hsa04310	Wnt signaling	✔			✔
GLI	hsa04340	Hedgehog signaling				
SMAD	hsa04350	TGF-β signaling				
PI3K	hsa04370	VEGF signaling				
PI3K	hsa04510	Focal adhesion	✔	✔		✔
PI3K	hsa04910	Insulin signaling	✔			
HIF1, PI3K, RTK	hsa05200	Pathways in cancer	✔			
HIF1	hsa05211	Renal cell carcinoma				

**Fig 7:**
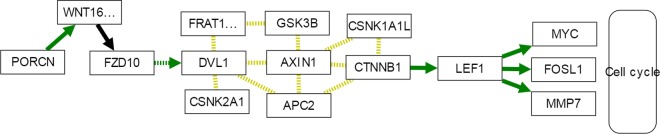
Part of the WNT signaling pathway for gastric cancer that shows the MinePath discriminant sub-paths. Green edges indicate discriminant functional relations for GC cases; edges in black indicate discriminant functional relations for both CG and normal cases; undirected yellow edges denote binding/association relations.

Fig 7 visualizes the discriminant functional sub-paths identified by MinePath in the Wnt pathway. As it can be observed, MinePath is in accordance with the specific outcome reported in [83], identifying the functional GC-related sub-path (indicated with the green colored edges), WNT16 (WNT5A) → FZD10 (FZD1) → DVL1 which, after a number of gene bindings/associations (engaging VANGL1,2) activates LEF1 which in turn activates MYC, FOSL1 and MMP7 to enter the Cell cycle pathway. Note also that MinePath was the only methodology that identified the P53 pathway as significant.

### MinePath on RNAseq data

**Fig 8:**
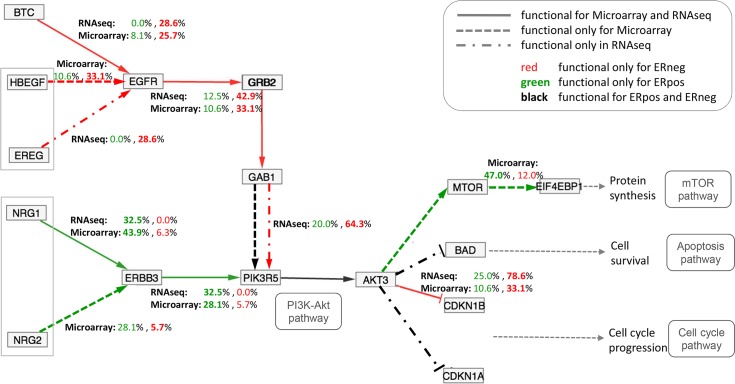
Contrasting between RNAseq and microarray gene expression profiling technologies on the pathway level

In order to validate and assess the ability of MinePath to cope with RNAseq gene expression data we applied it on the domain of BrCa targeting the ER phenotype (ER+ vs. ER-). The RNAseq data comes from a large scale multicenter BrCa study performed by the Sweden Cancerome Analysis Network—Breast (SCAN-B) Initiative [99] (GEO accession: GSE60788, 54 BrCa cases, 40 ER+ and 14 ER-). In addition, we applied MinePath on the ‘3ER GSE2034-3494-7390’ microarray (MA) gene expression dataset that was used for the validation of MinePath (section ‘ Self-assessment: MinePath for meta-analysis of gene expression studies’). Aiming to contrast between microarray and RNAseq gene expression measurements on the pathway level we focus on the ErbB pathway. The results are illustrated in Fig 8 where, different arrow types and colors are used in order to visualize the commonalities and similarities between the two dataset types.

Different types of arrows and colors are used in order to contrast between the relations and sub-paths identified as discriminant between the RNAseq and microarray datasets; drawing of the different types of lines was done manually in order to visually contrast between the two datasets. In general, the results from both RNAseq and microarray datasets are similar. For the ER- phenotype, the EGFR (epidermal growth factor receptor) is activated by the extra-cellular factors (direct arrow lines in red; see the explanatory legend at the top-right of the figure) BTC for both RNAseq and microarray, HBEGF for only microarray and EREG for only RNAseq in order to enter the intra-cellular regulation by the activation of GRB2, which in turn activates GAB1. The relation GAB1 → PIK3R5 is functional for both RNAseq (red dotted line) and microarray (black dashed line that is also functional for the ER- phenotype). Then, the functional sub-path continues with the common to the two datasets relation PIK3R5 → AKT3 and the inhibition of CDKN1B. Similar regulations hold for the ER+ phenotype (indicated by ‘green’ lines in Fig 8). It is notable that for both RNAseq and microarray datasets the cyclin-dependent kinase inhibitors CDKN1A and CDKN1B are inhibited and block the triggering of cell cycle events. A finding that is of interest concerns the ‘strength’ of the regulations identified as functional and discriminant for the two datasets. The AKT3—| CDKN1B inhibition relation covers 78.6% of the RNAseq ER- samples, compared with the respective 33.1% of the samples covered for the microarray dataset (the percentage figures are shown in Fig 8 over the inhibition relation). This may be suggestive for the superiority of RNAseq technology to measure RNA abundance more objectively because of its ability to detect low abundance transcripts and genes with higher fold-changes, as well as to avoid technical issues related to microarray hybridization [100]. The RNAseq experiment presents a first attempt towards the comparison between different gene expression profiling technologies on the level of molecular pathways, and at the same time it demonstrates the ability of MinePath to make such research quests feasible.

### MinePath and mutation-driven or post translational modifications

**Fig 9:**
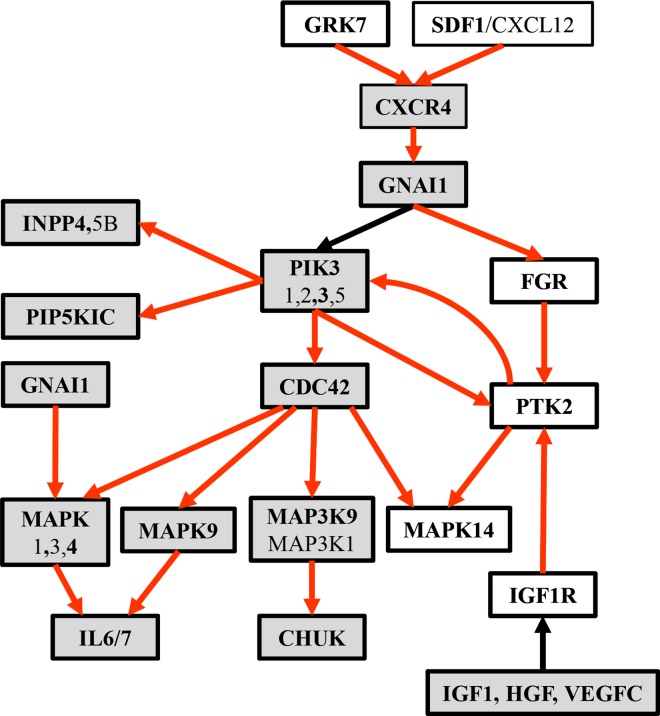
The integrated network that reflects the CXCR4 mutation downstream signalling events

Protein regulation can occur in both translational and post-translational level, and it is true that KEGG pathways engage and report both protein and expression changes (in the respective pathway maps and XML/KGML formatted files). As most gene set and pathway enrichment analysis approaches are based solely on gene expression measurements and data, they could not capture regulatory mechanisms that may not be reflected in gene expression data, such as post-translational modifications or kinetic control of biochemical reactions [101]. Quantitative relations due to differences in gene expression however, remain a strong indicator of protein regulation, and thus a useful tool for the identification of protein relations/regulation. Even though MinePath cannot directly detect post-translational modifications, the available information in KEGG pathways could be utilized for mapping differential gene expression and identification of relevant differential sub-paths. Under this setting, it remains a powerful tool for the identification of indicative and biologically significant relations. Moreover, as it is reported in a study about the connectivity of cancer co-expression networks, “… *the biological meaning of co-expression changes can be interpreted in terms of modifications of cancer genome landscape … that confirms the hypothesis that loss of connectivity fingers toward genes harbouring alterations (e*.*g*. *mutations*, *losses and deletions*, *promoter DNA methylation) or affected by post-translational modifications (e*.*g*. *phosphorylation*, *acylation*, *methylation*, *etc*.*) in tumors*.” matching of multi-dimensional data with samples for each kind of mutations is suggested in order to validate the hypothesis [102]. Under this driver, and on the basis of respective gene expression data, we assess the utility of MinePath in cases where mutations in an upstream regulatory factor can cause differential expression of target genes affecting their regulation. Assessment is based on the application of MinePath on a study that explores the principle role of the SDF1/CXCR4 axis in the homing and engraftment of hematopoietic stem/progenitor cells (HSPCs), with the proper functioning of CXCR4 downstream signaling to depend upon consistent optimal expression of both SDF-1 ligand and its receptor CXCR4 [103]. In this study, CXCR4 constitutive active mutations–CXCR4-CAMs (N119A and N119S) in K562 (human immortalized myelogenous leukemia) cell line were engineered. These CXCR4 mutations are able to induce autonomous downstream signaling in a regulated manner. To assess the effects of the specific CXCR4 mutations, the genome wide differential gene expression (microarray) profiles of three-(3) wild-type and six-(6) mutated samples were generated (with the Agilent-027114/Custom Human Whole Genome 8x60k Microarray; GEO accession: GSE76544). The CXCR4-CAMs resample the post translational modifications (PTMs) involved in the active state of the CXCR4 gene product. The task is to assess the ability of MinePath to identify and reveal potential regulatory relations and sub-paths caused by the corresponding gene expression alternations. It was encouraging to observe that most of these relations indeed affect gene targets that are downstream of CXCR4. The MinePath analysis results are illustrated in Fig 9 where, the downstream CXCR4 mutation signaling and the corresponding regulatory events (‘red’ colored edges) are mapped on an integrated regulatory network. The results are in accordance with the findings reported in the original study, most of the reported in the study paper CXCR4-mutation affected pathways are ranked as significant, with ‘MAPK’, ‘Phosphatidylinositol signaling’ and ‘Axon Guidance’ to be on the top of the reported MinePath list. In particular, and inspecting the network in Fig 9, the following observations could be made: (i) all the reported (in the study paper) genes are present in the network (shaded rectangular nodes), (ii) the non-shaded rectangles are genes (GRK7, FGR, PTK2, MAPK14, IGF1R, RASGRP1, RRAS2, RRAS) which are not reported in the study paper, and present putative targets for further research–especially the GRK7-CXCR4 axis is of interest for future studies, (iii) a positive regulation ‘loop’ between genes PTK2 and PIK3R3 is imprinted in the network (PTK2 and PIK3R3 are intracellular binding proteins involved in stromal contact in the bone marrow microenvironment [104]), a finding that is in accordance with a relevant comment in the study paper: “the differential gene expression profile of CXCR4 mutants reveals a positive loop of genes related to homing and engraftment”.

The aforementioned results and the integrated network in Fig 9 were produced by the following off-line analysis methodology: (i) the file of the differential sub-paths (as saved by MinePath) was processed, and all the single relations of each sub-path were extracted; (ii) a network with all the extracted relations was generated and imported in Cytoscape; (iii) using special functionality of Cytoscape the nodes/genes (and their synonyms) that are reported in the study paper were retained. The resulted integrated network, after rearranging its topology (view) layout, and renaming some of the nodes (to reflect their grouping in the KEGG pathways) is shown in Fig 9. The color of the edges follows the already represented coloring scheme, with ‘red’ and ‘green’ for the CXCR4-mutated and wild-type functional relations, respectively, and ‘black’ for relations which are functional for both cases. It is in our plans to automate and encompass the presented off-line analysis methodology in MinePath towards the creation of integrated networks with differential sub-paths that range across different pathways.

## Discussion

Integration of heterogeneous sources represents an effective venue, as compared to working within the boundaries of a single domain. This realization is particularly valid for the bioinformatics domain [105]. Bioinformatics and systems biology have demonstrated that knowledge across domains can better aid relevant scientific communities in their research. Pathway analysis methodologies that exploit the underlying regulatory machinery of pathways and the identification of phenotype differentiating sub-paths addresses and solves a typical problem of set enrichment strategies that is: the conflicting constrains between molecular pathways and gene expression data. An example is reflected in situations where two significantly up-regulated genes increase the enrichment of the set in gene expression data, even if one of the genes acts as an inhibitor of the other.

MinePath introduces a pathway analysis methodology that directly exploits the topology as well as the underlying pathway regulatory mechanisms, including the direction and the type of the engaged regulatory relations. This is in contrast with the traditional pathway analysis approaches that employ the so called Gene Set Analysis (GSA) [6] or Gene Set Enrichment Analysis (GSEA) [32] methodologies, with the target to identify the most significant (with respect to the target phenotypes) pathways. Even if there are some differences between the two methodologies (mainly with respect to the background statistical framework that they utilize) their fundamental characteristic is that they face pathways not as networks but just as groups (plain list) of associated genes. Both GSA/GSEA aim towards the reduction of differentially expressed gene lists (as assessed by gene ranking and selection) to biology relevant short lists that exhibit over-representation characteristics in targeted biological processes and molecular functions such as the ones present in pathways. Even in the lack of differentiating gene lists, GSA/GSEA may assist the identification of phenotype associated genes by taking advantage of the fact that many genes in a gene list may exhibit changes in their expression status under different functional conditions [106], and in some cases proved effective in improving predictive performance [15], [107]. Nevertheless, pathways are richer and encompass much more knowledge than just a plain list of genes, such as the topology and the involved gene regulatory relations recorded in the respective pathway networks. This important drawback of the GSA/GSEA approaches limits their ability to capture and model the multiple roles that genes take in the various molecular pathways. GSA/GSEA base their analysis on the cellular components (i.e., genes, proteins etc.) and not on the pathway networks’ connectivity (topology and interaction types) just because they compromise the underlying networks’ complexity in favor of computational simplicity [60]. Even if some of the existing pathway analysis methodologies and tools, like GSEA, take into account the topology and the underlying regulation machinery of pathways, a fundamental difference contrast them with the methodology followed by MinePath. The difference resides in the handling of the pathway gene regulatory relations. Most of these systems follow a scoring methodology in which, each regulatory relation is scored according to its status in the input gene expression data with activations to receive a ‘+1’ and inhibitions a ‘-1’ score, depending on their consistency with the respective gene expression sample profiles. A final score per sub-path is calculated and a final rank score per pathway is provided—an exception holds for GGEA, which provides sub-path qualitative consistency assessments. With such a ‘summation’ approach the risk to miss important regulations is increased (such a case is shown in the ‘Results’ section).

A final remark concerns the ability of MinePath to assess the phenotype differential power of pathway sub-paths and not the respective power of single regulatory relations. This unique feature of MinePath makes it a valuable tool for in silico molecular biology experimentation, and serves the biomedical researchers’ exploratory needs to reveal and interpret the underlying pathway regulatory mechanisms that putatively govern the expression of the target phenotypes.

The performance of MinePath was assessed using publicly available BrCa and CG gene expression data. The results demonstrate the validity of the MinePath methodology in devising sub-path based predictive models. It would be of interest to compare and contrast the predictive performance of MinePath with the performance of traditional differential gene expression analyses, as well as the degree of overlapping genes between different datasets and phenotypes. Such a comparison is out of the scope of the current paper, as the main focus is on the detailed presentation of MinePath sub-path based pathway analysis methodology and its comparison with relative state-of-the-art pathway analysis methodologies. A fair comparison with traditional gene expression analyses methodologies will require a large enough collection of diverse gene expression datasets as well as different (algorithmic) parameterization arrangements, and it is in our plans to set-up and conduct such a systematic and large-scale assessment study.

The comparison of MinePath with state-of-the-art pathway analysis methodologies like SPIA, GGEA, DAVID, GSEA and PATHOME highlights the value of the system, not only for its ability to identify important molecular regulations but also for its web-based implementation as well as, for its interactive visualization capabilities that facilitates the biological interpretation of the findings. Using a meta-analysis approach on three merged BrCa ER datasets, and focusing on the well-known ErbB signaling pathway, we provided indicative evidence for the power of MinePath to identify and reveal important molecular cancer-related regulatory operations that governs the expression of specific BrCa phenotypes. Although protein regulation can occur in both translational and post-translational level, quantitative relations of gene expression still remain a strong indicator of protein regulation, and thus a useful tool for the identification of protein relations/regulation. As an example, MinePath was able to reveal the downstream effects and the corresponding regulatory machinery that underlies CXCR4-mutant affected genes).

MinePath is in active continuous development with ongoing work and planned extensions to target and include: (i) automation of the CXCR4-mutant analysis methodology in order to create integrated networks with differential sub-paths that range across different pathways; (ii) support multi-class gene expression data in order to differentiate between more than two target phenotypes–exploiting relevant research from the machine learning field, transformation of a multi-class problem to different two-class/binary problems seems a promising direction to follow–optimization approaches are also of relevance [108], and will be assessed for their customization to the MinePath methodology; (iii) adaptation of more pathway databases and relative pathway representation formats (except from KEGG)–such an extension will enable the assessment of the robustness of results across different pathway databases; (iv) offer services for automated uploads of gene expression data repositories (e.g. from Gene Expression Omnibus (GEO) and TCGA/Cancer Genome Atlas Research Network (cancergenome.nih.gov)); (v) provide more enriched annotations and respective links for the visualized results (genes, relations, pathways etc)–such an extension will ease the users to focus their inquiries on specific genes of interest (e.g., genes that belong to particular molecular function), and will enable the respective filtering and restriction of the input gene expression profiles just to these genes and (vi) visualization of differential genes, e.g., gene signatures from various studies, could be also supported (a first attempt, to be included as a stable component of MinePath, is implemented in the application of MinePath on a study that concerns the determination of the biological relevance of transcription factor binding sites over functional pathway sub-paths [109]).

## Materials and Methods

The overall MinePath methodology encompasses and implements five modular components: (i) discretization of gene expression data; (ii) decomposition of pathways into their constituent sub-paths; (iii) matching and assessment of the functional status of sub-paths in gene expression sample profiles, (iv) assessment of the phenotype differential power of the decomposed sub-paths; and (v) visualization of the results. MinePath generates a set of informative and phenotype differentiating pathway sub-paths that uncover the molecular regulatory mechanisms, and putatively govern the expression of targeted phenotypes. The web-based MinePath environment offers a rich visualization framework where the user may adjust the differentiating power of targeted pathways according to his/her research exploratory needs.

### Discretization of gene expression values

In gene expression studies, the quest is not only to identify genes that differentiate between phenotype classes but also to uncover putative correlations between these genes. Such an approach seems most reasonable in pathway analysis, in the sense that gene biomarkers need to be mapped to some biological function. As biochemical reactions are governed by discrete events (at least at the lowest reaction levels–oxidation, reduction, catalysis etc.) it is reasonable to consider gene expression values by a ‘qualitative’ manner–with the underlying assumption that mRNA transcriptomic measurements capture and represent abundance sufficiency in a binary mode, i.e., “*Is the gene up-regulated or down-regulated*?” “*Is the gene expressed or not*? “*Is the gene expression high or low*?”. As it is noted by Hartemink in his thesis on the discovery and validation of gene regulatory networks [110], discretization of gene expression values shows a number of advantages. First, it allows modelling of qualitative relationships between genes while reducing considerably the domain dimensionality. Second, discrete state representation of gene expressions approximate regulatory transcriptional equilibria can be captured and represented by qualitative statements. Third, under rational (domain dependent) assumptions the discretization introduces a measure of robustness against error, including errors in gene expression measurements and their normalization. Fourth, at one extreme, discretized gene expression values present approximations to the reported continuous ones and at the other, continuous gene expression measurements present an approximation of the underlying discrete processes and events within the cells. Biologically driven discretization of gene expression values could be done on the presence and knowledge of some experimentally determined control value—the respective continuous values are assigned and transformed into respective binary representatives depending whether they are lower or greater than the respective control [111]. On the absence of such knowledge that is normally difficult to be objectively determined and assessed, automated and statistically driven approaches are followed. Discretization of gene expression values is already followed in many microarray studies and respective data analysis approaches [112], [113].

Among the various discretization methods, the supervised Entropy-based global discretization approach (ED in short) gained a lot of attention. In a global data pre-processing mode, ED follows a top-down splitting process examining all putative split-points which then are iteratively re-split to incrementally form the respective discretization intervals [114]. One of the most inspiring and well-defined method is presented by Fayyad and Irani in [115]. For a given set of sample cases *S*, and a feature *A* the method chooses (in an iterative top-down mode) a cutting point *T*_*A*_ (selected among the class borderlines) in a way that the joint class information entropy *E(T*_*A*_*;S)* is minimized. A similar approach is also introduced by Li and Wong [116]. Experimental results on benchmark datasets for various discretization approaches showed that the Fayyad and Irani ED discretization method is by far the best performing method [117]. In addition, in [118] is shown that ED binary discretization does not suffer from data fragmentation and so, no significant accuracy degradation occurs.

**Fig 10:**
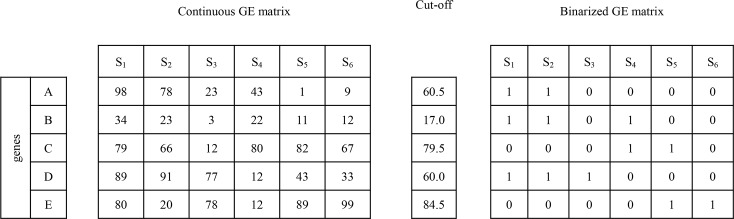
Discretization of gene expression values in MinePath. At the left a ‘dummy’ gene expression profile is shown, the profile refers to five genes (rows) and to six samples (columns); at the right its discrete binarized version of the gene expression profile is shown; in between the respective (for each gene) computed discretization cut-off points are shown.

MinePath follows and implements an ED binary discretization process in order to transform gene expression values into high (expressed / up-regulated) or low (not-expressed / down-regulated) gene expression binary equivalents. Our method resembles the Fayyad & Irani and Li & Wong approaches. Even if both methods employ entropy-based statistics they do not incorporate an explicit parameter to force binary splits, a fact that may result to uncontrolled numbers of discretization intervals (more than two), which would be difficult to interpret in the presence of two phenotype classes. Below we give a general description of the MinePath discretization process that unfolds into four steps [119]:

1. The expression values of a gene over the total number of input samples are sorted in descending order;
2. The midpoints between each two consecutive values are calculated;
3. For each midpoint, μ_i_, the Information Gain (*IG*) of the system is computed—*IG* offers a way to optimally differentiate (with the gene at hand) between sample cases taking into account their prior phenotype class assignment, and it is mainly used by decision-tree induction algorithms [120]. Let *IG(S*,*μ*_i_*)* to denote the *IG* of the system for midpoint μ_i_,
  $$IG(S,\mu_{i})=E\left(S\right)–E(S,\mu_{i})$$
  $$E(S)=-\sum_{i}{P(C}_{i},S)*log({P(C}_{i},S))$$
  where, *E(S)* is the entropy of the system taking into account the prior assignment of sample cases into phenotype classes, *P(C*_*i*_,S) the proportion of samples in S that belong in Class C and *E(S*,*μ*_*i*_*)* the respective entropy of the system taking into account its division into subgroups around midpoint μ_i_. The midpoint with the highest information gain is selected as the discretization point.
4. The sample cases with expression values lower than the discretization point are assigned the ‘0’ value (meaning that the gene is under-expressed), and the sample cases with expression values bigger that the discretization point are assigned the ‘1’ value (the gene is over-expressed). The discretization process is applied for each gene separately, and the final dataset is a matrix of discretized, actually binarized, values. Fig 10 illustrates the MinePath discretization process using a ‘dummy’ gene expression dataset.

Every probability distribution is trivially a maximum entropy probability distribution under the constraint that the distribution have its own entropy. Entropic formulas are applicable not only to uniform distributions but also to Poisson or negative binomial distributions (as a mixture of Poissons) [121]. Hence, the MinePath entropy based discretization is appropriate and applicable also to RNAseq data since it is known that RNAseq data normally follow Poisson or negative binomial distributions [122].

For a reliable and effective pathway analysis methodology, both the pathways and the gene expression data have to use the same nomenclature. Pathways use gene ids (KEGG uses Entrez gene IDs) while gene expression platforms use probeset ids. The mapping from a gene nomenclature and thesaurus to another rises the many-to-one issue where, many probesets are assigned to the same Entrez /KEGG gene ID. Multiple probesets targeting the same gene do not (should not) show different expression levels’. Thus, taking into account the expression status of just one of the probes would be enough. Since we cannot assure consistency between the different microarray platforms, MinePath checks the multiple probesets for each gene and infers a combined expression value by applying a logic OR between the respective probeset values. This is actually the same as selecting the probe value that exhibits the highest intensity out of all the probes that map to the same gene.

### Functional sub-paths: Matching sub-paths with gene expression profiles

A molecular pathway is considered as a graph with nodes to represent genes, groups of genes and compounds, and edges to represent regulatory gene relations such as activation, inhibition, expression, indirect, phosphorylation, dephosphorylation, ubiquitination, association and dissociation. Each pathway is decomposed into all of its constituent sub-paths following a depth-first search approach, implemented as a ‘find all’ function which is able to identify all paths from a node to every other node. As an example consider the four (artificial) genes A, B, C and D and an artificial pathway consisting by just one path: A → B → D—| C. The pathway is decomposed into six sub-paths, the single-relation sub-paths A → B, B → D, and D—| C; the overlapping sub-paths A → B → D and B → D—| C; as well as the original pathway (considered as a sub-path) A → B → D—| C.

After the decomposition of each a pathway into its constituent sub-paths, each sub-path is matched against the input binary gene expression sample profiles. The functional status of a sub-path in a sample is assessed by a set of binary (Boolean) operations and a set of semantics that decipher the exact molecular nature of the involved gene relations. Table 6 summarizes the types of KEGG relations and the respective semantics that MinePath employs for their modeling. Activation is modelled and implemented with the AND Boolean operator, meaning that A → B is functional in a sample if and only if both A and B are up-regulated. In all other cases the activation relation is considered as non-functional. Inhibition possesses a dual interpretation which is modelled by the XOR (exclusive OR) Boolean operator. So, A—| B is considered as functional either if A is up-regulated and B is down-regulated or, if A is down-regulated and B is up-regulated, in the sense that gene B is allowed to be up-regulated and active because its inhibitor A is down-regulated. There are relevant studies that adopt such a dual interpretation of inhibition [123]. In a more theoretical framework, the effect of an inhibiting gene depends on its level of expression: when it is up-regulated it has a negative effect, i.e., down-regulates its targets, and when it is down-regulated it holds a positive effect, i.e., up-regulates its targets [124]. MinePath considers ‘Binding/Association’ and ‘Dissociation’ as special pathway relations that do not exhibit a specific regulatory effect but as a condition in which specific genotypes are associated with other factors [125]. Therefore, MinePath identifies and visualizes binding/association and dissociation relations independently of the expression status of the engaged genes.

**Table 6:** Semantics of pathway gene relations in MinePath ‘UP’, ‘DOWN’ stand for up-regulation and down-regulation of genes, respectively.

Relation	Truth Table				MinePath Semantics
Activation			**B**		If A is UP then B is UP
**A→B**			UP	DOWN	
	**A**	UP	✔	✕	
		DOWN	✕	✕	
Inhibition			**B**		If A is UP then B is DOWN or if A is DOWN then B is UP
**A⊣B **			UP	DOWN	
	**A**	UP	✕	✔	
		DOWN	✔	✕	

Expression					As Activation
**A→o→B**					
Indirect effect					As Activation
**A**─ **→B**					
Phosphorylation					
**A**^**+p**^**→B**					As Activation
**A**^**+p**^**⊣B **					As Inhibition
De-Phosphorylation					
**A**^**-p**^**→B**					As Activation
**A**^**-p**^**⊣B **					As Inhibition
Ubiquitination					
**A**^**+u**^**→B**					As Inhibition
Association/Binding			B		
**A—B**			UP	DOWN	
Dissociation	**A**	UP	✔	✔	
**A-|-B**		DOWN	✔	✔	

**Fig 11:**
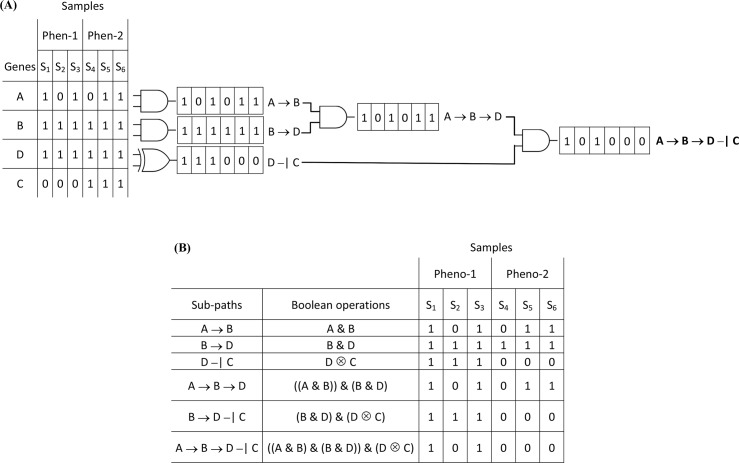
Identification of functional sub-paths in gene expression sample profiles (matching operation). (A) Identification of functional sub-paths and their matching with gene expression profiles; a ‘dummy’ binary gene expression profile is used with four genes and six samples assigned to two phenotype classes; ‘1’ represents up-regulated and ‘0’ down-regulated statues of gene, respectively. (B) The binary sub-path expression matrix produced by MinePath.

MinePath copes with two basic single gene regulatory relations, activation/expression and inhibition, encoded by the AND and XOR Boolean operators, respectively. For more complex sub-paths MinePath takes into account the binary value of the path’s last relation and the binary value of the sub-path’s part examined so-far, and combines them with an AND operator. Fig 11A illustrates the matching operation based on a ‘dummy’ binary gene expression profile. Consider the artificial sub-path A → B → D—| C. Initially, and using the AND operator, the truth-value of A → B is assessed for each truth binary value in each of the input samples; if A and B are both up-regulated (i.e., both takes the value ‘1’ in the discretized binary gene expression matrix) the relation is considered as functional in the sample. The same process is followed in order to assess the binary value of A → B → D. That is, the binary values of A → B and B → D are combined using again the AND operator. Then, the resulting binary values of A → B → D and D—| C are combined, using again the AND operator, to form the final binary value of the input sub-path. Note that the matching process computes not only the binary value of the input sub-path but the respective binary values of all overlapping sub-paths as well (including the sub-path’s single-relations).

The final outcome is the sub-path (binary) expression matrix, just in analogy with traditional gene expression matrix, with rows all the decomposed pathway sub-paths (including the overlapping ones), columns the input samples, and each cell to get the values ‘1’ or ‘0’ for the functional status of the corresponding sub-path in the respective sample (Fig 11B). For the example of Fig 11, the sub-path A → B → D—| C is functional for cases S_1_ and S_3_, the sub-path A → B → D is functional for S_1_, S_3_, S_5_ and S_6_, and D—| C is functional in all Phenotype-1 and non-functional in all Phenotype-2 samples. The B → D—| C sub-path exhibits the highest phenotype differential power as it perfectly discriminates between the two phenotypes.

### Identification of the phenotype differential power of sub-paths

The sub-path expression matrix does not aim to reduce the dimensionality of gene expression profiles. In fact, it may involve more sub-path features than the number of genes present in the original input gene expression matrix. For this reason, and in analogy to gene selection, MinePath encompasses and implements a multi-parametric sub-path selection process for the identification of the most discriminant sub-paths. The selection process is implemented by the employment of feature-selection and classification techniques (see below). MinePath saves the Weka version of the sub-path expression matrix that contains just the most significant and selected sub-paths. The user may then input this file to Weka in order to induce a variety of predictive models in the form of decision trees, support vector machines, Naïve Bayes classifiers etc.

On the complete sub-path dataset, a set of various filters are applied in order to exclude non-informative sub-paths and select the most significant of them. This process acts as a general filtering approach and may be used independently as a generic gene expression pre-processing strategy. Filtering has been demonstrated to be very useful for p-value adjustment and information elucidation [126]. MinePath encompasses three distinct filters in order to assess the phenotype differential power of sub-paths and select the most discriminant from them namely, coverage, p-value and polarity with a respective configurable threshold to each one of them.

#### Coverage

The percentage of samples (for a specific phenotype) in which the sub-path is functional should be at least 25%.

#### p-value

MinePath uses the Fisher exact test or the D. Benjamini & Hochberg False Discovery Rate (‘B&H adjusted fdr’) [127] (as a default option in the MinePath user interface) in order to compute a two-tailed p-value or adjusted p-value for each sub-path and assess its significance. Sub-paths that exhibit a p-value less than 0.05 pass the filter. The formula used to compute the p-value of each sub-path is:

$$p-value=\frac{\left(\frac{a+b}{a})(\frac{c+d}{c}\right)}{\left(\frac{a+b+c+d}{a+c}\right)}=\frac{(a+b)!(c+d)!(a+c)!(b+d)!}{(a+b+c+d)!a!b!c!d!}$$

where, *a* and *b* are the number of phenotype-1 and phenotype-2 samples in which the sub-path is functional, respectively; *c* and *d* the number of phenotype-1 and phenotype-2 samples in which the sub-path is non-functional, respectively. The D. Benjamini & Hochberg False Discovery Rate is computed using the following formula:

$$B\&H\;adjusted\;fdr=p-value*\frac{m}{i}$$

where *m* is the number of all sub-paths and *i* is the rank of the sub-path in the ascending order of the respective p-values.

#### Polarity

The formula below computes the polarity rank, *r*_*sb*_, for each sub-path; the metric measures the power of the sub-path to distinguish between the two target phenotypes (*a*, *b*, *c* and *d* as above):

$$r_{sb}=\frac{\frac{a}{a+c}-\frac{b}{b+d}}{\frac{a}{a+c}+\frac{b}{b+d}}$$

The polarity formula provides positive values for sub-paths that are functional mainly in phenotype-1 samples, and negative values for sub-paths that are functional mainly in phenotype-2 samples. The sub-paths that exhibit absolute polarity value higher than 0.5 are considered discriminant (a user defined threshold that could be tuned in the MinePath user interface).

Only the sub-paths that pass all the respective filters are selected and retained as most discriminant. Permutation tests using FDR (as described in section ‘Self-assessment: Robustness analysis via permutation testing’) prove that MinePath provides low false positive rates and delivers robust results.

Here we have to note that even though the sub-path polarity metric is independent from the sample size, and can be effectively utilized for small (per target phenotype) sample sizes, the sub-path p-value, and especially the ‘B&H adjusted fdr’ estimate cannot be used with small sample sizes. It is known that most of the differential gene expression analyses require a minimum of five to eight samples per class for a two classes dataset [128], [129]. This presents just a minimum but not optimum requirement [130]. Even if a minimum of five samples per class is required for reliable p-value calculations, the differential expression of sub-paths may be assessed using less samples if we opt to ignore the significance implications (p-value) of the sub-paths (this option available only for the standalone version of MinePath).

In addition, MinePath identifies those sub-paths that are almost always functional for both phenotypes (e.g., over 90% of the samples for each phenotype, also a user tuned threshold). Such sub-paths possess no phenotype differential power but are important in order to fill-in the gap between two discriminant sub-paths, and enable the formation of long sub-paths that reveal biologically relevant routes in pathways.

The filter-based assessment of the differential power of sub-paths does not aim to infer causal or even phenotype-associated sub-paths. It just infers a set of highly predictive sub-paths. The causal or associative interpretation of the selected sub-paths requires further investigation and a subsequent downstream analysis.

#### Assessing the significance of pathways as a whole

MinePath assess also the significance and ranks the pathways according to their p-values. The pathway p-value is calculated based on a more strict variation of the Fisher exact test, similar to the EASE score proposed by DAVID (david.ncifcrf.gov/content.jsp?file=functional_annotation.html—fisher) [126]. The difference is that MinePath identifies functional and discriminant sub-paths instead of genes, and this make the normal EASE score inappropriate as we face the problem of overlapping sub-paths, e.g., the path A → B—| C → D is decomposed into six sub-paths, even if it involves just three gene regulatory relations. So, we run the risk of high numbers of functional sub-paths that leads to too low p-values. For this reason, MinePath computes the (one-tailed) pathway p-value taking into account the unique single-relations involved in the functional sub-paths, forming and utilizing the contingency matrix shown in Table 7. Note that, even though the EASE formula is the same for MinePath and DAVID, MinePath uses counts for the single relations involved in the identified functional and differential sub-paths, while DAVID uses differential genes while DAVID uses counts of discriminant genes.

**Table 7:** Contingency table for pathway p-value calculation For each pathway *p*, the quantities *a*_*p*_ and *b*_*p*_ represent the number of functional single relations (as part of functional sub-paths) and the total number of relations in the pathway, respectively; the quantities *c*_*p*_ and *d*_*p*_ represent the corresponding quantities in all other pathways.

	Functional relations	Total relations
In pathway	*a*_*p*_	*b*_*p*_
Not in pathway	*c*_*p*_	*d*_*p*_

Based on the aforementioned contingency matrix, MinePath uses the formula below to compute the p-value of each pathway.

$${p-value}_{p}=\frac{\left(\frac{{(a}_{p}-1)+b_{p}}{a_{p}-1})(\frac{c_{p}+b_{p}}{c_{p}}\right)}{\left(\frac{n}{{(a}_{p}-1)+c}\right)}$$

The p-value formula takes in consideration all the pathway functional single relations (as part of functional sub-paths) regardless of their phenotype inclination–*a*_*p*_ is the sum of functional relations in either phenotype-1 or phenotype-2 samples. So, it presents an estimate of “*how much functional the pathway is*” and not “*the differential power of the pathway*”. In other words, MinePath follows and implements an enrichment pathway-analysis approach, just in analogy with GSEA approaches, with a critical difference that highlights the MinePath paradigm shift in pathway analysis. That is, instead of looking for gene set enrichment features, MinePath assesses the sub-path set enrichment characteristics of a pathway, taking in consideration the functional status of the single relations of the decomposed pathway functional sub-paths. This approach has the following influential effects: (a) the risk to consider equivalent pathways with the same genes is avoided, (b) the underlying (functional) regulatory machinery of the pathways is fully reflected in the assessment of pathway significance, and (c) the significance of a pathway gets a more natural meaning in terms of the number of functional relations and sub-paths used to differentiate between the target phenotypes–an effect that could be further exploited by the MinePath visualization operations and services. The final result of MinePath is a p-value ranked list of pathways from which the user may select a pathway to visualize and explore.

### Functionality and visualization capabilities of MinePath

MinePath identifies and visualizes the differentially expressed sub-paths utilizing and appropriately customizing the Cytoscape graph visualization Web library (js.cytoscape.org). In addition, the system supports active interaction and re-adjustment of the visualized network as it is equipped with special network editing capabilities that enable the reduction of pathways’ complexity. To the best of our knowledge MinePath is the only tool that visualizes differentially expressed pathway relations and sub-paths instead of differential genes, and this is done with a color-coding schema that reflects the phenotype inclination of relations.

The MinePath web-server is a Web 2.0 application. It relies on the frontend-backend software design using AJAX calls for the communication. The layout, appearance and interface of the front-end have been implemented in JavaScript. For the visualization of pathways the Cytoscape.js library is deployed and expanded. The MinePath backend is implemented as a java-based application.

In its current version MinePath supports 299 KEGG signaling pathways, from which the user may select/deselect as the input target pathways for further analysis. The system provides also a number of stored gene expression profiles (mainly from the GEO repository) but the user may format and upload its own gene expression profile (relative help material is provided to assist the user to form and format custom gene expression datasets). Optionally, the user may adjust various parameters such as the minimum threshold percentage of sub-paths that are functional for both phenotypes (default value is set to 90%), the minimum polarity rank threshold to decide if a sub-path is discriminant or not (default value is set to 0.5), the optional use of p-value or ‘B&H adjusted fdr’ as the threshold (sub-paths with p-values more than 0.05 are ignored), and fold change as a threshold to filter sub-paths (only the sub-paths that exhibit at least a two-fold change between the two target phenotypes are retained).

**Fig 12:**
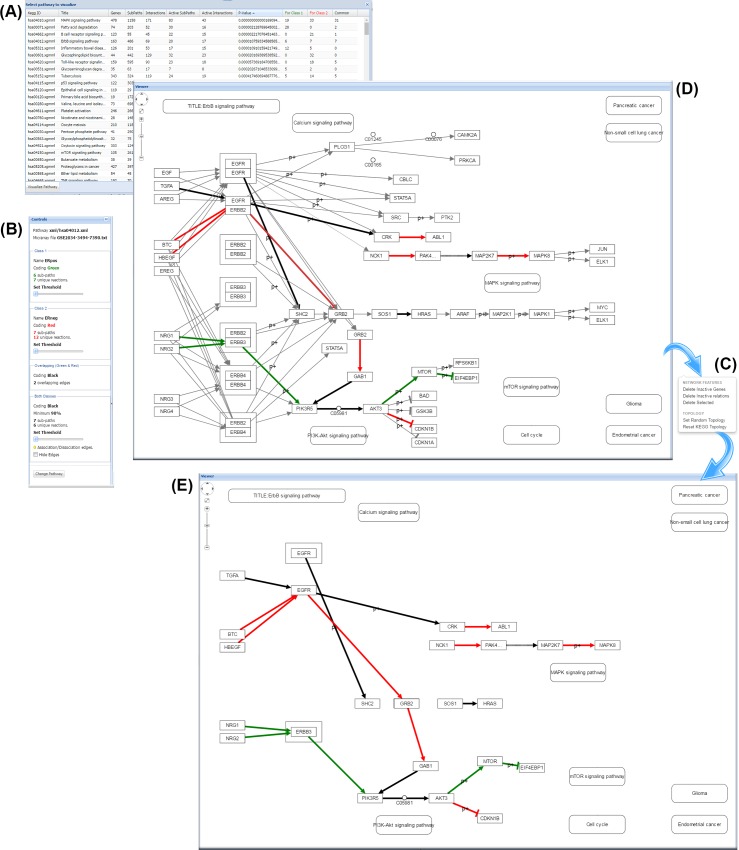
MinePath pathway visualization functionality and capabilities. (A) Sorted (by p-value) list of pathways accompanied by other statistics computed by MinePath–the user may select the pathway to visualize. (B) MinePath ‘Controls’ panel. (C) MinePath pathway editing panel. (D) The original ErbB pathway with its KEGG layout preserved. (E) The edited and simplified ErbB pathway

One of the key features that distinguish MinePath from other pathway analysis methodologies and tools rests in the system’s visualization functionality, and especially in the visualization of functional gene regulatory relations and sub-paths that differentiate between the target phenotypes (see Fig 12). MinePath visualizes differentially expressed relations. It also retains the KEGG topology layout of the pathway, a layout that illustrates the underlying biology of the network, ease its exploration and give insight into the underlying molecular regulation mechanisms of the pathway (see the ErbB pathway shown in Fig 12D). The gene regulatory relations are mapped with different colors in order to reveal their phenotype inclination: green for relations being functional in phenotype-1 samples; red for relation being functional in phenotype-2 samples; black for relations considered as functional in both phenotypes; As an example consider the sub-path A → B → C that is found to be functional for phenotype-1, and sub-path X → A → B → Y to be functional for phenotype-2. The common sub-path A → B is shared by both phenotypes and so, the activation ‘→’ relation is colored in black. Grey color is used for non-functional and yellow for associations/dissociation relations. MinePath is equipped with special interactive visualization functionality that enables the reduction of network’s complexity and re-orientation of its topology. Moreover, in MinePath there are links for each of the visualized gene (by clicking on it) to the respective gene and drug-related KEGG sections as well as to PharmGKB (www.pharmgkb.org) relevant gene variants and associated drugs, as a first approach to offer putative clinical utilization of the system. With the ‘Controls’ panel (Fig 12B) the user may dynamically set (via sliders) stricter thresholds for the most discriminant sub-paths to display (based on their polarity score). Via a pop-up menu (Fig 12C) the whole pathway may be easily simplified (e.g., delete genes and non-functional relations), edited, re-shaped and re-arranged; Fig 12E shows the simplified version of the original ErbB pathway shown in Fig 12D. The list of all pathways is displayed in a special window (Fig 12A), sorted according to their p-values (or other metrics) from which the user may select the pathway to visualize.
